# Recombinant Slit2 Reduces Surgical Brain Injury Induced Blood Brain Barrier Disruption via Robo4 Dependent Rac1 Activation in a Rodent Model

**DOI:** 10.1038/s41598-017-00827-z

**Published:** 2017-04-07

**Authors:** Prativa Sherchan, Lei Huang, Onat Akyol, Cesar Reis, Jiping Tang, John H. Zhang

**Affiliations:** 1 Department of Physiology and Pharmacology, Loma Linda University, Loma Linda, California 92354 USA; 2 Department of Anesthesiology, Loma Linda University, California, 92354 USA; 3 Department of Neurosurgery, Loma Linda University, California, 92354 USA

## Abstract

Brain tissue surrounding surgical resection site can be injured inadvertently due to procedures such as incision, retractor stretch, and electrocauterization when performing neurosurgical procedures, which is termed as surgical brain injury (SBI). Blood brain barrier (BBB) disruption due to SBI can exacerbate brain edema in the post-operative period. Previous studies showed that Slit2 exhibited vascular anti-permeability effects outside the brain. However, BBB protective effects of Slit2 following SBI has not been evaluated. The objective of this study was to evaluate whether recombinant Slit2 via its receptor roundabout4 (Robo4) and the adaptor protein, Paxillin were involved in reducing BBB permeability in SBI rat model. Our results showed that endogenous Slit2 increased in the surrounding peri-resection brain tissue post-SBI, Robo4 remained unchanged and Paxillin showed a decreasing trend. Recombinant Slit2 administered 1 h before injury increased BBB junction proteins, reduced BBB permeability, and decreased neurodeficits 24 h post-SBI. Furthermore, recombinant Slit2 administration increased Rac1 activity which was reversed by Robo4 and Paxillin siRNA. Our findings suggest that recombinant Slit2 reduced SBI-induced BBB permeability, possibly by stabilizing BBB tight junction via Robo4 mediated Rac1 activation. Slit2 may be beneficial for BBB protection during elective neurosurgeries.

## Introduction

Neurosurgical procedures can inadvertently cause damage to brain tissue surrounding the surgical site, which is termed as surgical brain injury (SBI)^1–4^. Disruption of the blood brain barrier brain (BBB) is a major pathophysiological consequence following neurosurgical procedures which worsens post-operative brain edema and neurological function^5–8^. Increase BBB permeability contributes to perisurgical site brain edema following neurosurgical injury in experimental SBI rodent model^9, 10^. Furthermore, disruption of BBB junction proteins increases paracellular permeability leading to the development of subsequent vasogenic edema which can worsen patient’s post-operative neurological outcomes^11^. Current treatment regimens such as diuretics, steroids, and osmotic agents used for post-operative brain edema are non-specific, do not target specific BBB pathology, and have limited use due to unwanted adverse effects^1, 12, 13^. Safe therapeutic interventions that target BBB specific pathology may be beneficial to reduce post-operative brain edema.

There are 3 known isoforms in the Slit family of proteins (Slit1–3)^14, 15^. Among the members of the Slit proteins, Slit2 which is a secreted extracellular matrix protein is known to be involved in regulating migration of axons and neurons during development^14, 16^. Recent studies show that Slit2 increased in the brain following traumatic^17^ and surgical brain injury^18^, which implicates that Slit2 may have a role to play during recovery after brain injury in adults^16^. Slit proteins bind to the immunoglobulin type receptors Roundabout (Robo1–4) which initiates intracellular signal transduction pathways that modulate various cellular functions^15, 19^. Among the Robo receptors, Robo4 has been shown to be an endothelial specific receptor^20^. Slit2 and Robo4 receptor has been implicated to regulate endothelial function and vascular permeability. In a model of glioma cocultured endothelial cells, exogenous Slit2 pretreatment reduced blood tumor barrier (BTB) permeability which was inhibited with knockdown of Robo4 receptor^21^. Likewise, recombinant Slit2 reduced vascular hyperpermeability in a Robo4 receptor dependent manner in mouse models of retinopathy^22^ and lung inflammation^23^. Furthermore, the endothelial stabilizing effect of Robo4 has been shown to be mediated by the downstream intracellular adaptor protein, Paxillin^24^.

The role of Slit2 in regulating BBB permeability after SBI has not been explored. We proposed that recombinant Slit2 administration will activate Robo4-Paxillin signal transduction pathway which will attenuate SBI induced BBB disruption and improve outcomes in a rat model.

## Methods

### Animals

Animal procedures followed NIH Guide for the Care and Use of Laboratory Animals. The study protocol was approved by Loma Linda University Institutional Animal Care and Use Committee (IACUC). Adult Sprague Dawley rats (n = 70), male, 280–350 g, were randomly divided into Sham or SBI groups. Animals were kept in a facility with controlled humidity and temperature, 12 h light/dark phase, and given food ad libitum.

### Experimental Design and Animal Groups

#### Experiment 1

The expression of endogenous Slit2, Robo4 and Paxillin at various time-points following SBI was evaluated. The groups included Sham and SBI groups at 6 h, 12 h, 24 h and 72 h after injury. The peri-resection right frontal lobe samples were obtained for western blot to evaluate temporal expression of the proteins and immunohistochemistry was performed for cellular localization of Robo4 and Paxillin.

#### Experiment 2

The BBB protective effect of recombinant Slit2 was assessed. The groups included Sham, SBI + Vehicle, SBI + Slit2 (10 µg/Kg). Rats were injected recombinant Slit2 (R and D Systems, Minneapolis, MN) or normal saline as vehicle via intraperitoneal route 1 h prior to inducing SBI^18, 25^. Rats were subjected to neurological testing 24 h post-SBI and then euthanized to collect peri-resection right frontal lobe samples for western blot to evaluate BBB junction proteins and for Evans blue dye extravasation assay to evaluate BBB permeability.

#### Experiment 3

The role of Robo4 and Paxillin in BBB protective effect of recombinant Slit2 was assessed. The groups included Sham, SBI + Vehicle, SBI + Slit2 (10 µg/Kg), SBI + Slit2 (10 µg/Kg) + Robo4 siRNA, SBI + Slit2 (10 µg/Kg) + Paxillin siRNA, SBI + Slit2 (10 µg/Kg) + Scramble siRNA. Rats were injected recombinant Slit2 (R and D Systems, Minneapolis, MN) or normal saline as vehicle via intraperitoneal route 1 h prior to inducing SBI^18, 25^. The siRNA for Robo4, Paxillin and Scramble siRNA (all from Life Technologies, Grand Island, NY) was injected via intracerebroventricular (ICV) route 24 h prior to SBI. Neurological testing was performed 24 h post-SBI and peri-resection right frontal lobe samples were collected for western blot and to measure Rac1 activity.

### Surgical Brain Injury Rat Model

The rat surgical brain injury model was made as described previously^1, 10^. Anesthesia was induced with 4% isoflurane and kept at 2.5% during surgery. A midline incision was made on the scalp and skull was exposed. A craniotomy 5 × 5 mm was performed in right side frontal bone with margins 2 mm lateral to sagittal suture and 1 mm proximal to coronal suture. Bone flap was removed to expose the underlying dura and right frontal lobe. Right frontal lobe was partially resected following margins of the bone window. The depth of resection was extended to base of skull. After resection was completed, the cavity was irrigated with normal saline followed by intra-operative packing to ensure hemostasis. Skin incision was sutured once complete hemostasis was achieved. Sham surgery included right frontal craniotomy but dura and frontal lobe was kept intact. Post-operative analgesia was provided using buprenorphine (0.03 mg/Kg) injected subcutaneously at the end of surgery. Rats were monitored at regular intervals post-operatively and returned back to home cages after complete anesthetic recovery.

### Intracerebroventricular Injection

Intracerebroventricular (ICV) injection was performed under isoflurane anesthesia as previously described^26^. Rats were positioned prone on a stereotactic frame. The following coordinates were used to make a burr hole on the right parietal bone: 1 mm lateral and 1.5 mm posterior in relation to bregma, and 3.2 mm depth from the surface as described previously^26, 27^. A total volume of 2 μL either Robo4 siRNA (500 pmol) or Paxillin siRNA (500 pmol) (Life Technologies, Grand Island, NY) was infused 0.5 μL/min into lateral ventricle using a 10 µL Hamilton syringe (Hamilton Co, Reno, NV) through the burr hole as described previously^26, 27^. Scramble siRNA (Life Technologies, Grand Island, NY) 2 μL was infused in the negative control group. The needle was kept in position for 10 mins after infusion ended and removed over 5 mins to avoid backflow. Bone wax was used to seal the burr hole and the skin was sutured. Rats were monitored post-operatively and returned back to home cages after anesthetic recovery. The siRNAs for both Robo4 and Paxillin were reconstituted in nuclease free water for delivery as provided by the vendor and carrier was not used. To improve knockdown efficacy, two different sequences targeting Robo4 were pooled: Robo4 (i) sense, 5′-GACUACGAAUUCAAAGUGAtt-3′; antisense, 5′-UCACUUUGAAUUCGUAGUCtt-3′; (ii) sense, 5′-CAGCCUCAGUAGUCGACUAtt-3′; antisense, 5′-UAGUCGACUACUGAGGCUGct-3′. Additionally, two different sequences targeting Paxillin were pooled: Paxillin (i) sense, 5′-GCUUCAUUGUCAGAUUUCAtt-3′; antisense, 5′-UGAAAUCUGACAAUGAAGCca-3′; (ii) sense, 5′-GGACAACCCUACUGUGAAAtt-3′; antisense, 5′-UUUCACAGUAGGGUUGUCCgt-3′.

### Neurological Evaluation

The modified Garcia test was performed to evaluate sensorimotor deficits 24 h after SBI in a blinded manner as previously described^10, 28, 29^. Briefly, the test evaluated seven parameters: activity in cage, proprioception, whisker touch response, symmetry of limbs, turning, outstretching of forepaws, and climbing. Each parameter received a score ranging from 0 or 1 upto 3 to get a total score ranging from 3 to 21. Higher scores indicated better performance.

### Evans Blue Dye Extravasation

Evans blue dye extravasation assay was performed 24 h after surgery to assess BBB permeability as previously described^1, 26^. Briefly, Evans blue dye (2%; 5 mL/kg) was given by intraperitoneal injection and allowed to circulate for 4 h after injection. During sacrifice, transcardial perfusion of phosphate buffered saline (PBS) was performed after which the brain samples were removed, snap frozen in liquid nitrogen and stored at −80 °C until use. The peri-resection right frontal samples was homogenized in PBS (1 mL/300 mg) and then centrifuged at 14,0000 rpm for 30 mins after which the supernatant was collected. An equal amount of trichloroacetic acid (50%) was added to 500 μL of the supernatant and allowed to incubate overnight at 4 °C. The supernatant was centrifuged using the same parameters next day. The extravasation of Evans blue dye was measured at 620 nm with a spectrophotometer. The extravasated Evans blue in brain samples was quantified using a standard curve and expressed as micrograms per gram of brain tissue as previously described^30–32^.

### Western Blot Analysis

Western blot protocol was followed as described previously^33, 34^. During sacrifice, rats were perfused through the heart with PBS and brain samples were obtained. Ripa lysis buffer (Santa Cruz Biotechnology, Dallas, TX) was used to homogenize the right frontal lobe samples and then centrifuged at 14,000 g for 30 mins at 4 °C. Supernatants were collected and protein concentration was measured. Samples (50 µg) were loaded to sodium dodecyl sulfate polyacrylamide gel for electrophoresis then transferred to nitrocellulose membrane. Following primary antibodies were applied on to membranes and incubated at 4 °C overnight: anti-Slit2 (1:500), anti-Robo4 (1:3,000), anti-Paxillin (1:200) (all from Santa Cruz Biotechnology, Dallas, TX), anti-occludin (1: 50,000) and anti-claudin3 (1:1,000) (both Abcam, Cambridge, MA), and anti-VE cadherin (1:200) (Santa Cruz Biotechnology, Dallas, TX). Membranes were incubated with anti-β Actin (1:2,000) (Santa Cruz Biotechnology, Dallas, TX) as loading controls. Secondary antibodies (1:2,000) (Santa Cruz Biotechnology, Dallas, TX) were applied on to membranes for 1 h at room temperature. The ECL Plus Chemiluminescence reagent (Amersham Biosciences, Arlington Heights, IL) was applied to visualize the bands. Density of the bands was quantified using Image J software (National Institutes of Health, Bethesda, MD). Relative density of bands was calculated as ratio to β-actin and then normalized to average of Sham group as described previously^30^. Detailed information of the antibodies used for western blot experiments are listed in Supplementary Table 1.

### Immunohistochemistry

Briefly, rats were perfused with PBS and formalin after which the brain samples were collected and stored in formalin. Using a cryostat (CM3050S; Leica Microsystems, Bannockburn, IL), 10 µm thick sections were obtained. Immunofluorescence staining procedure was performed as described previously^33, 34^. The following primary antibodies were applied onto sections and incubated at 4 °C overnight: anti-Robo4 (1:100) or anti-Paxillin (1:100) (both from Santa Cruz Biotechnology, Dallas, TX) and co-localized with anti-von willibrand factor (vWF) (1:100) (Santa Cruz Biotechnology, Dallas, TX), anti-glial fibrillary acidic protein (GFAP) (1:100) or anti-neuronal nuclear protein (NeuN) (1:100) (both from Abcam, Cambridge, MA). Next, appropriate FITC- and Rhodamine Red-conjugated secondary antibodies (1:100) (Jackson Immuno Research, West Grove, PA) were applied at room temperature for 2 h. A fluorescence microscope (Olympus BX51) was used to visualize the sections.

### Rac1 Activity Assay

A Rac1 activity kit (Cell Biolabs, San Diego, CA) was used to evaluate Rac1 activity as described previously^35, 36^. PAK1-PBD agarose beads was added to right frontal lobe samples and incubated for 1 h at 4 °C to pull-down active Rac1 in samples. Samples were centrifuged to isolate the beads. Sample buffer was added to the bead samples followed by electrophoresis using 10% polyacrylamide gel and nitrocellulose membrane transfer. Anti-Rac1specific antibody from the kit was applied to membranes and incubated at 4 °C overnight. Films were developed after applying appropriate secondary antibody for 2 h at room temperature to detect Rac1-GTP.

### Statistical Analysis

Data were expressed as mean ± SEM, and analyzed using Sigma Plot 10.0 and Sigma Stat 3.5 (Systat Software, San Jose, CA). One-way ANOVA for multiple comparisons and Student-Newman-Kuels post-hoc test was used to analyze differences among groups. P value less than 0.05 was taken as statistically significant.

## Results

### Time course expression of Slit2 post-SBI

**Figure 1:**
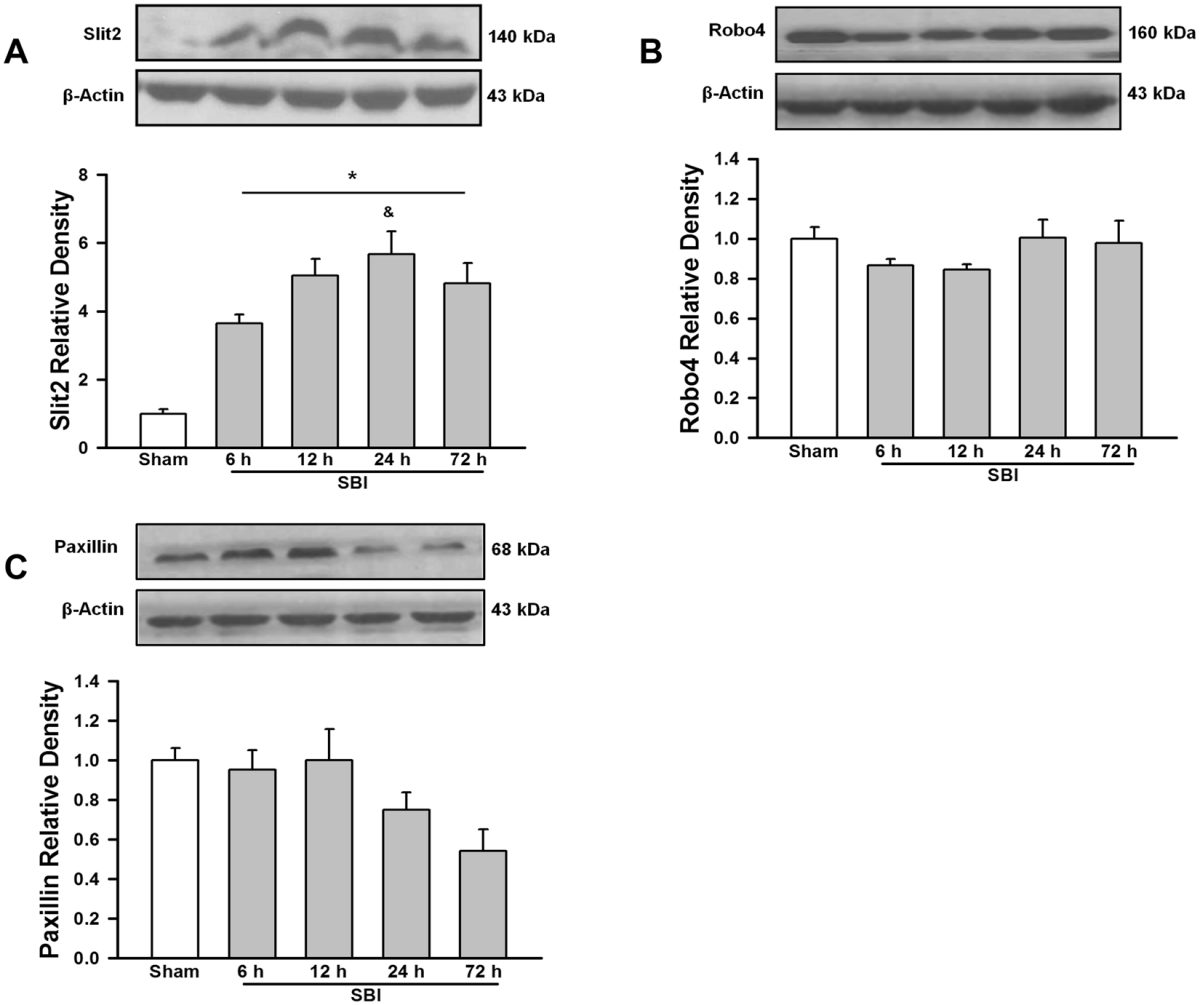
Temporal expression of endogenous Slit2, Robo4 and Paxillin in the right frontal perisurgical site after SBI. (**A**) Representative western blot showed that the expression of endogenous Slit2 increased in the right frontal perisurgical region at various time points after SBI. Data are expressed as mean ± SEM. ANOVA, SNK. N = 4/group. *p < 0.05 compared to Sham, ^&^p < 0.05 compared to SBI 6 h. (**B**) The expression of Robo4 did not change at the right frontal perisurgical site up to 72 h after SBI. Data are expressed as mean ± SEM. ANOVA, SNK. N = 4/group. (**C**) Western blot showed a trend in reduced expression of Paxillin at 24 and 72 h after SBI at the right frontal perisurgical site. Data are expressed as mean ± SEM. ANOVA, SNK. N = 4/group. The full length blots for western blot pictures shown in panels A, B, and C are presented in Supplementary Figure S1.

Slit2 expression in the peri-resection right frontal lobe samples was measured using western blot analysis of brain samples collected at 6 h, 12 h, 24 h, 72 h after SBI. There was an increase in the expression of Slit2 at all time points post-SBI compared to sham group (p < 0.05; Fig. 1A).

### Time course expression and cell types expressing Robo4 post-SBI

**Figure 2:**
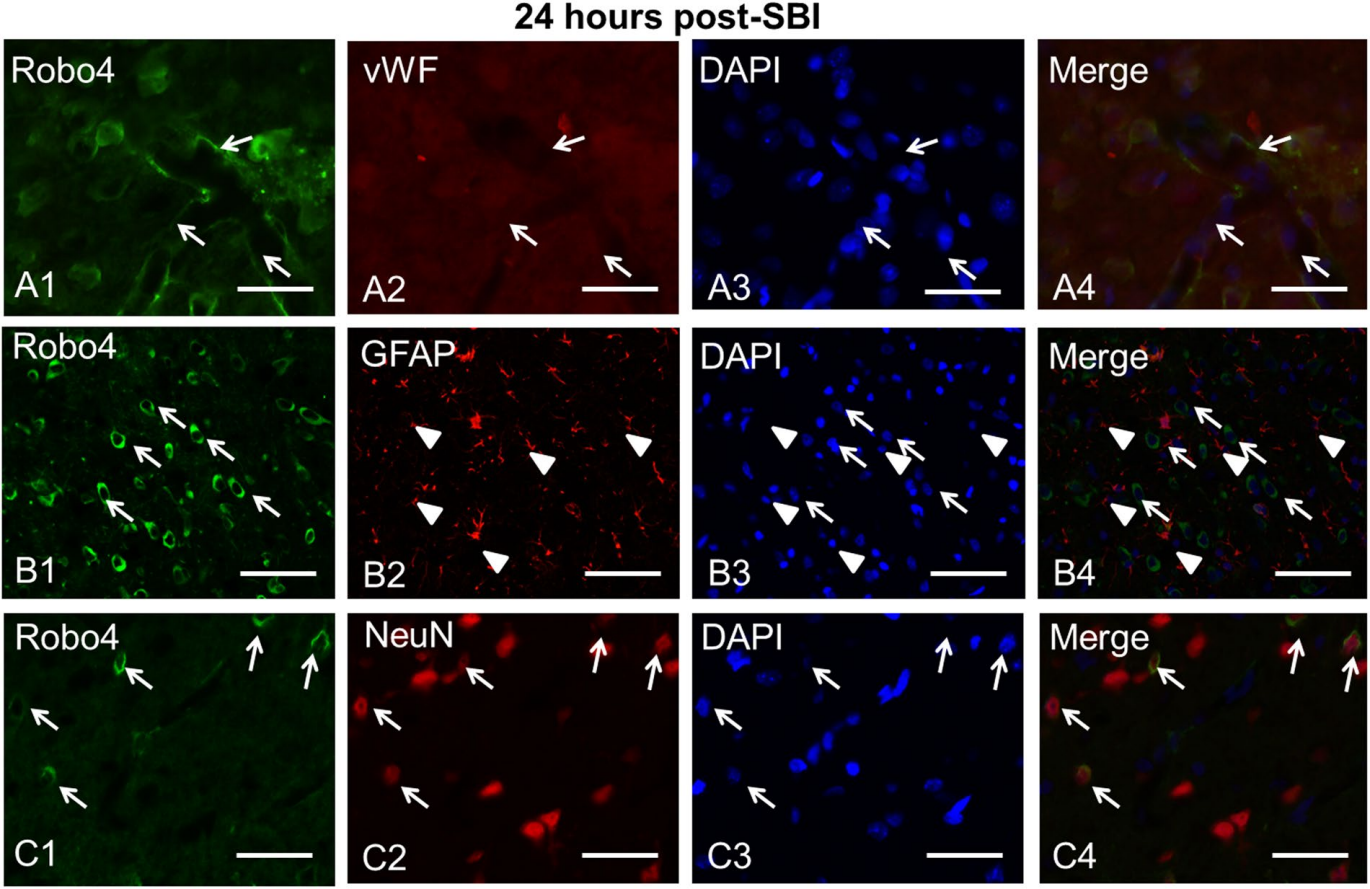
Localization of Robo4 in the brain at the right frontal perisurgical site 24 h after SBI. (**A**) Representative microphotographs of immunofluorescence staining showed co-localization of Robo4 (FITC/green) with the endothelial marker von Willibrand Factor (vWF) (Rhodamine Red/red) and DAPI (blue). (**B**) Immunofluorescence staining showed Robo4 (FITC/green) was expressed by neurons but did not co-localize with the astrocyte marker glial fibrillary acidic protein (GFAP) (Rhodamine Red/red). (**C**) Immunofluorescence staining showed Robo4 (FITC/green) co-localized with neuronal marker NeuN (Rhodamine Red/red) and DAPI (blue) at the perisurgical site 24 h after SBI. Arrows and arrowheads indicate cells with positive staining. Scale bar = 50 μm.

Robo4 expression in the peri-resection right frontal lobe samples was measured using western blot analysis at 6 h, 12 h, 24 h, 72 h after SBI. There was no change in the expression of Robo4 post-SBI compared to sham group (p > 0.05; Fig. 1B). Right frontal lobe samples obtained after SBI were stained with double immunofluorescence staining to delineate cell types that express Robo4. The endothelial cells (Fig. 2A) and neurons (Fig. 2C) expressed Robo4 but not astrocytes (Fig. 2B) in the peri-resection tissue 24 h post-SBI.

### Time course expression and cell types expressing Paxillin post-SBI

**Figure 3:**
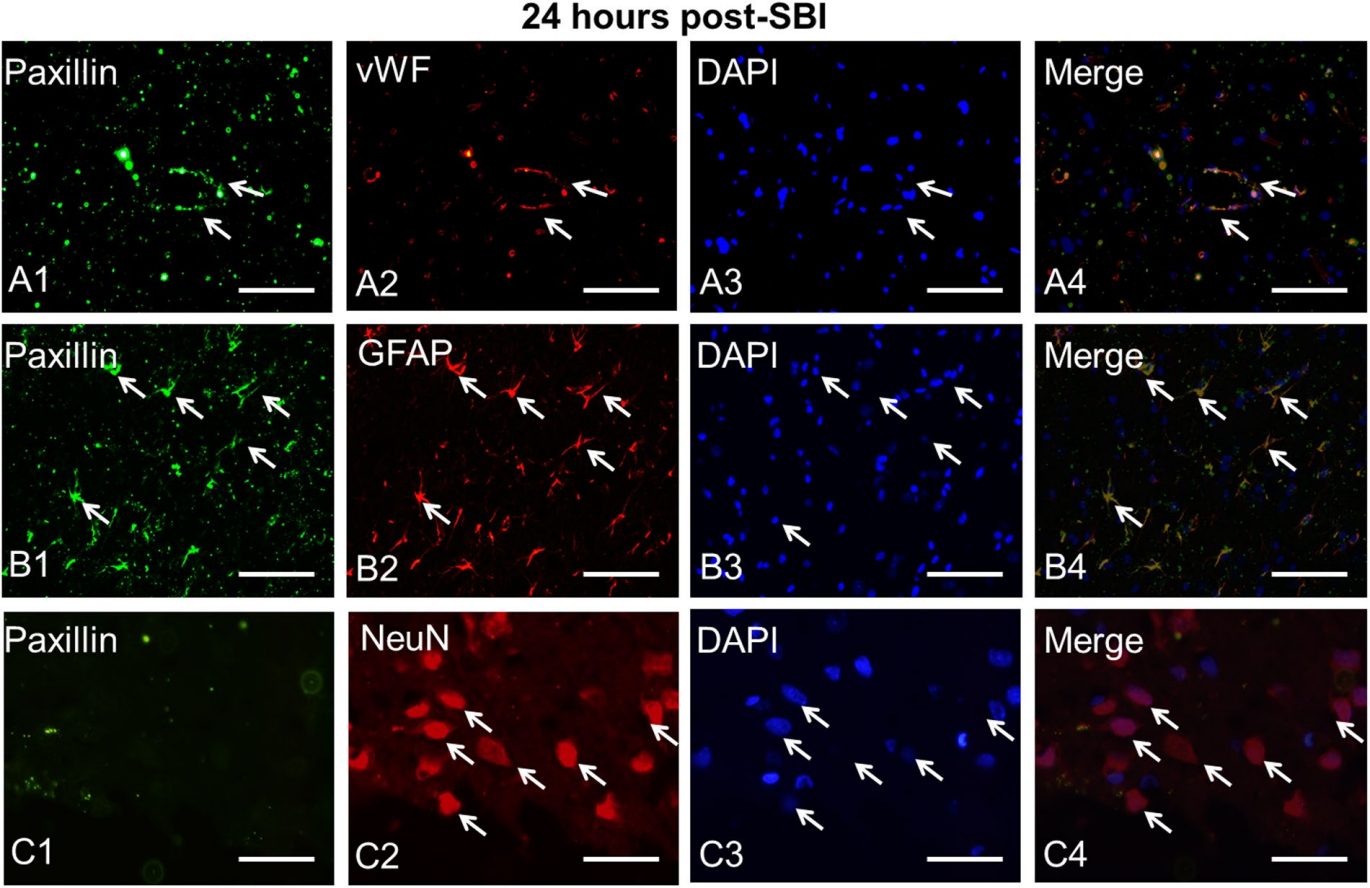
Localization of Paxillin in the brain at the right frontal perisurgical site 24 h after SBI. (**A**) Representative microphotographs of immunofluorescence staining showing co-localization of Paxillin (FITC/green) with von Willibrand Factor (vWF) (Rhodamine Red/red), and DAPI (blue). (**B**) The pictures show co-localization of Paxillin (FITC/green) with GFAP (Rhodamine Red/red), and DAPI (blue). (**C**) Immunofluorescence staining showed Paxillin (FITC/green) did not co-localize with neuronal marker NeuN (Rhodamine Red/red). Arrows indicate cells with positive staining. Scale bar = 50 μm.

Paxillin expression in the peri-resection right frontal lobe samples was measured using western blot analysis at 6 h, 12 h, 24 h and 72 h after SBI. The expression of Paxillin was not significantly different from sham post-SBI (p > 0.05; Fig. 1C). However, Paxillin showed a decreasing trend 24 h and 72 h post-SBI. Double immunofluorescence staining showed that Paxillin co-localized with endothelial cells (Fig. 3A) and astrocytes (Fig. 3B) but not with neurons (Fig. 3C) in the peri-resection tissue 24 h post-SBI.

### Recombinant Slit2 administration preserved BBB tight junction protein expression 24 h post-SBI

**Figure 4:**
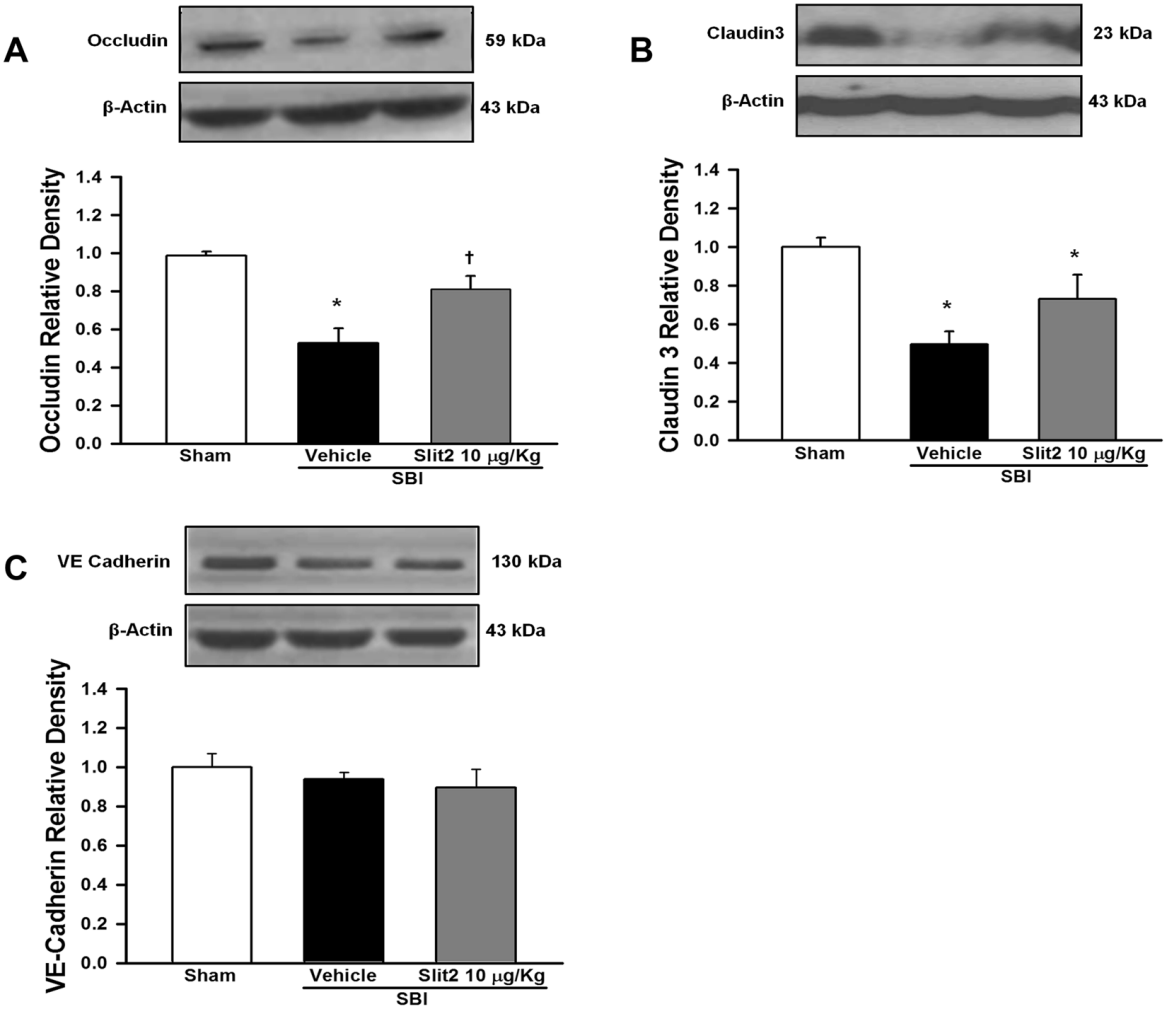
Effect of recombinant Slit2 on BBB junction protein expression 24 h after SBI. (**A**) Representative western blot analysis showed recombinant Slit2 (10 μg/Kg) administration significantly increased the expression of occludin after SBI. (**B**) The expression of claudin 3 showed a trend to increase after SBI with recombinant Slit2 (10 µg/Kg). (**C**) The expression of VE cadherin did not change after SBI. Data are expressed as mean ± SEM. ANOVA, SNK. N = 4–6/group. *p < 0.05 compared to Sham, ^†^p < 0.05 compared to SBI + Vehicle. The full length blots for western blot pictures shown in panels A, B, and C are presented in Supplementary Figure S2.

The BBB junction proteins occludin, claudin3 and VE cadherin in the peri-resection right frontal samples was evaluated using western blot analysis 24 h post-SBI. Occludin and claudin3 expression was reduced significantly post-SBI compared to sham group (p < 0.05; Fig. 4A and B, respectively). Recombinant Slit2 administered SBI group had significantly increased expression of occludin compared to vehicle group (p < 0.05) and a trend to increase the expression of claudin3 post-SBI. However, the expression of VE cadherin was not significantly different between sham or SBI groups at 24 h (p > 0.05; Fig. 4C).

### Recombinant Slit2 administration attenuated BBB permeability and neurological deficits 24 h post-SBI

**Figure 5:**
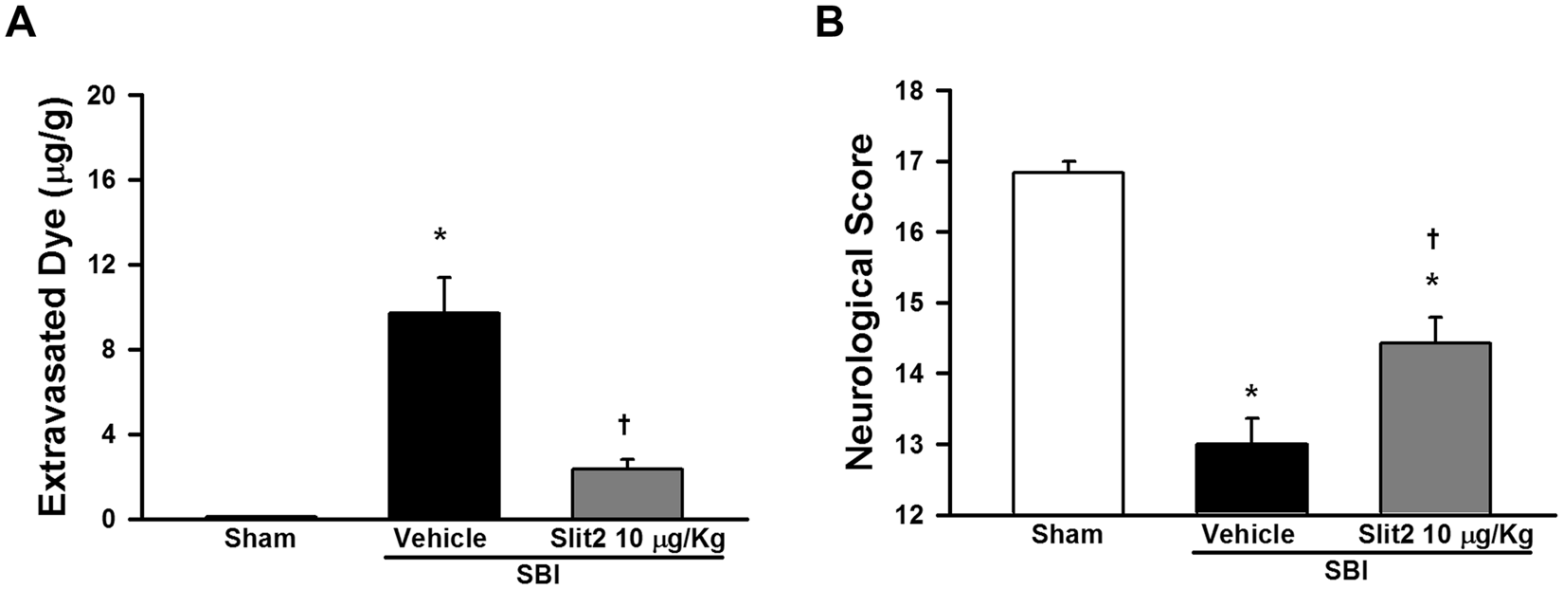
Effect of recombinant Slit2 on BBB permeability and neurological function 24 h after SBI. (**A**) Evans blue dye extravasation in the right frontal perisurgical site was reduced by recombinant Slit2 (10 μg/Kg). (**B**) Modified Garcia test showed recombinant Slit2 (10 µg/Kg) administration improved neurological score 24 h after SBI. Data are expressed as mean ± SEM. ANOVA, SNK. N = 6–7/group. *p < 0.05 compared to Sham, ^†^p < 0.05 compared to SBI + Vehicle.

The permeability of BBB was evaluated using Evans blue dye extravasation assay at 24 h after SBI. Rats subjected to SBI had significantly increased extravasated dye in the peri-resection right frontal tissue compared to sham group (p < 0.05; Fig. 5A). Recombinant Slit2 administration reduced significantly the extravasation of Evans blue dye post-SBI compared to vehicle group (p < 0.05). Recombinant Slit2 administered SBI group had significantly lower neurological deficits, which was observed as increased neurological scores evaluated by modified Garcia test 24 h post-SBI (p < 0.05; Fig. 5B).

### Recombinant Slit2 administration increased Rac1 activity 24 h post-SBI dependent of Robo4-Paxillin pathway

**Figure 6:**
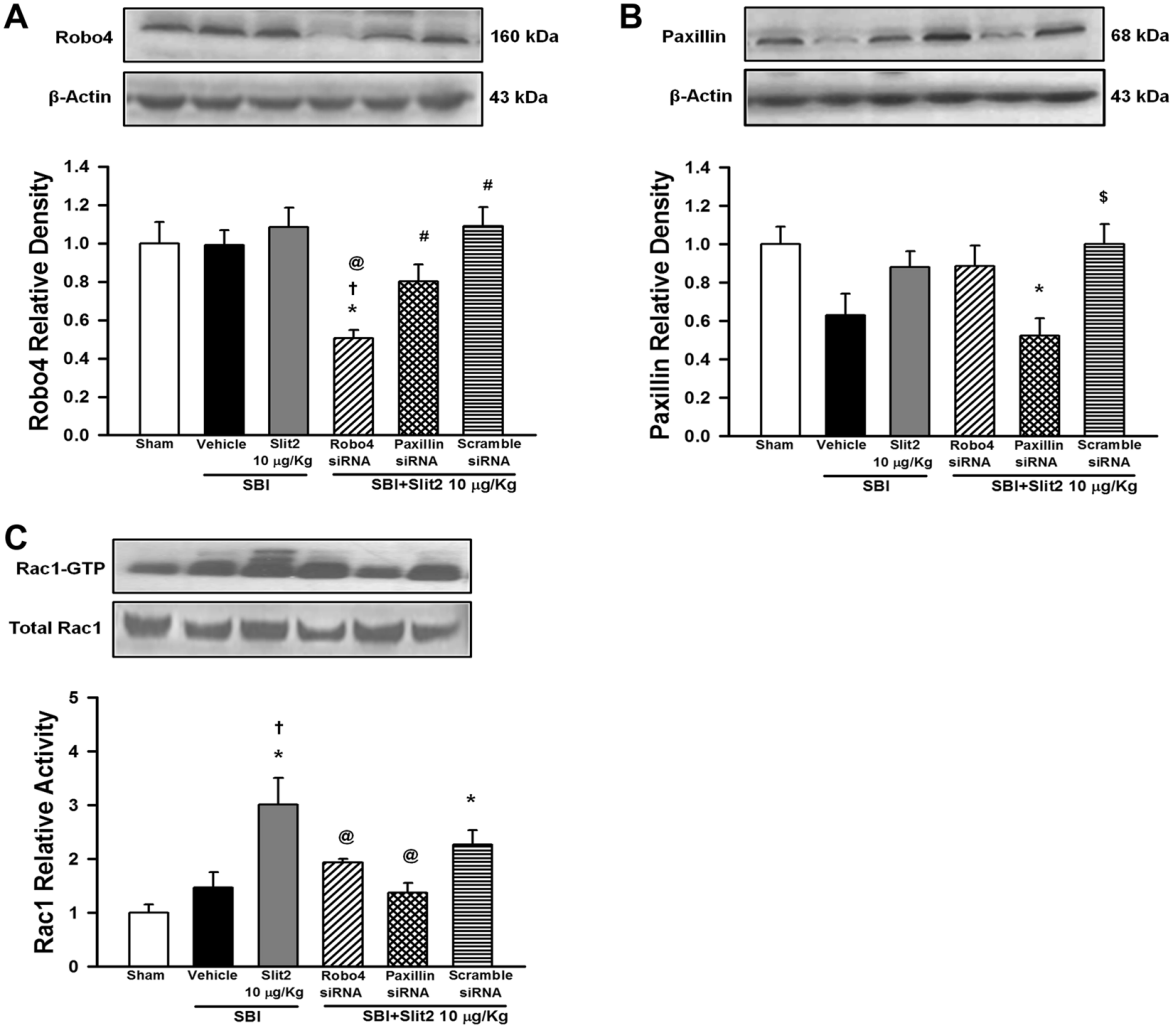
Role of Robo4-Paxillin pathway in Slit2 mediated protection after SBI. (**A**) Representative western blot analysis at 24 h after SBI to show the efficacy of Robo4 knockdown using siRNA. The expression of Robo4 did not change after SBI or with recombinant Slit2 (10 μg/Kg) administration compared to Sham. The expression of Robo4 was significantly reduced with Robo4 siRNA but not with Paxillin siRNA or scramble siRNA. (**B**) Paxillin siRNA but not scramble siRNA significantly reduced Paxillin expression. (**C**) Rac1 activity assay showed recombinant Slit2 (10 μg/Kg) administration increased Rac1 activity 24 h after SBI. Robo4 siRNA and Paxillin siRNA reversed this effect but not scramble siRNA. Data are expressed as mean ± SEM. ANOVA, SNK. N = 4/group. *p < 0.05 compared to Sham, ^†^p < 0.05 compared to SBI + Vehicle, ^@^p < 0.05 compared to SBI + Slit2 10 μg/Kg, ^#^p < 0.05 compared to SBI + Slit2 + Robo4 siRNA, ^$^p < 0.05 compared to SBI + Slit2 + Paxillin siRNA. The full length blots for western blot pictures shown in panels A and B, and Rac1 activity assay shown in panel C are presented in Supplementary Figure S4.

The assay for Rac1 activity was performed at 24 h after SBI with the administration of Robo4 or Paxillin siRNA along with recombinant Slit2. Robo4 siRNA administration with recombinant Slit2 reduced significantly the expression of Robo4 24 h post-SBI compared to Paxillin siRNA or scramble siRNA administration (p < 0.05; Fig. 6A). Paxilin siRNA administration with recombinant Slit2 decreased significantly the expression of Paxillin compared to scramble siRNA administration (p < 0.05; Fig. 6B). Recombinant Slit2 administration significantly increased Rac1 activity 24 h post-SBI compared to vehicle group that was reversed by Robo4 siRNA and Paxillin siRNA (p < 0.05; Fig. 6C) but not scramble siRNA administration (p > 0.05).

## Discussion

This study examined the BBB protective effects of recombinant Slit2 administration in a rat SBI model. Our findings showed that recombinant Slit2 reduced SBI induced BBB permeability which was associated with the preservation of endothelial tight junction protein expression. Robo4 receptor and Paxillin was expressed by endothelial cells at the right frontal peri-resection tissue post-SBI. SBI rats that received recombinant Slit2 showed increased Rac1 activity post-SBI that was reversed with Robo4 siRNA and Paxillin siRNA administration along with recombinant Slit2. Our findings suggest that recombinant Slit2 attenuated BBB disruption after SBI, possibly via stabilizing tight junction protein through Robo4-Paxillin dependent activation of Rac1 after SBI.

We first evaluated the endogenous Slit2 expression at various time-points post-SBI. Our findings showed that Slit2 started increasing at 6 h following SBI and remained elevated till 72 h following injury. Slit2 has been shown to be expressed by various cell types in the brain including neurons, astrocytes, and endothelial cells^37–39^. Previous studies from our lab and others have shown that Slit2 increased in response to an insult in the brain including traumatic brain injury (TBI)^17^, SBI^18^ and cerebral ischemia^39^. Additionally, our previous study showed that endogenous Slit2 had a protective function after SBI^18^. These finding suggest that Sli2 has protective role following brain injury. We therefore sought to examine whether recombinant Slit2 administration would augment protection after SBI. Specifically, in this study we explored the BBB specific protective function of recombinant Slit2 in a rat SBI model.

Slit2 is known to function by binding to Robo receptors. Among the Robo family receptors, Robo4 is known to be the endothelial specific receptor for Slit2 ligand^14, 20^. Robo4 receptor has been demonstrated to be expressed by endothelial cells^20^ including human brain microvascular endothelial cells^21^. Consistent with these findings, we observed that Robo4 was expressed by endothelial cells at the perisurgical site in SBI rats. Interestingly, we also found that Robo4 was expressed by neurons but not astrocytes surrounding the perisurgical site. Additionally, our results showed that the expression of Robo4 evaluated 24 hours after SBI did not change compared to sham group. In a transient cerebral ischemia rat model, Robo4 weakly increased at day 3 and continued to increase progressively at days 14 and 28 after reperfusion and was expressed by reactive astrocytes in the CA1 region of hippocampus^39^. It is likely that expression of Robo4 is induced in the long term after injury during reactive astrogliosis. It is also possible that Robo4 may be differentially expressed in varying pathologies. Previous study showed that the expression of Robo4 was upregulated in human glioma tissues compared to normal brain tissue^21^. Furthermore, Robo4 was shown to be abundantly expressed by endothelial cells in various tumor samples^40^. In a tumor environment, Robo4 has been implicated to regulate the migration of endothelial cells during tumor angiogenesis^40^. Since Robo receptors are known to mediate their function via activation of intracellular signal transduction pathways, we suggest that in the presence of recombinant Slit2 the endothelial protective Robo4 signaling pathway gets activated despite no change in receptor expression after SBI. The regulation of Robo4 expression after SBI remains to be further explored.

Paxillin is an intracellular adaptor protein that binds to structural and signaling molecules^24, 41^. It functions as a scaffold protein to which signaling molecules dock and activate intracellular signal transduction pathways^42^. Previous study showed that Paxillin was a critical downstream effector of Robo4 receptor in endothelial cells^24^. Furthermore, Paxillin was shown to play a key role in endothelial barrier enhancement in human lung microvascular endothelial cells^43^. We therefore investigated if Paxillin played a role in BBB stabilization by recombinant Slit2. First, we evaluated the expression of Paxillin after SBI. We observed that Paxillin was expressed by endothelial cells and astrocytes at the perisurgical site but not by neurons. Additionally, we observed a decreasing trend in the expression of Paxillin after SBI despite a strong expression of Paxillin noted in astroctyes. The decrease in Paxillin after SBI therefore may be more selective for the vasculature and neurons. Since we used whole brain samples taken from the perisurgical site which includes all cell types for western blot experiments, this may partly explain the decreasing trend in Paxillin expression observed after SBI despite a strong expression seen in astrocytes. Various factors such as extracellular matrix proteins, tyrosine kinases, cytokines^44^, growth factors^45^, lipopolysaccharide^41^ have been reported to regulate Paxillin. These factors have been shown to increase after SBI and may negatively regulate Paxillin following injury^9, 10, 34, 46^.

Although primarily involved in regulating migration of developing axons and neurons^16, 19^, recent studies have demonstrated that Slit2 can exert its effects on other cells types including endothelial cells and regulate endothelial barrier integrity^21, 47, 48^. Several studies have shown that Slit2 protected endothelial integrity. Slit2 maintained endothelial barrier stability of human lung lymphatic endothelial cells exposed to HIV *in vitro* ^47^. Additionally, VEGF-induced endothelial hyperpermeability *in vitro* and in mouse models of retinal and choroidal vascular disease was reduced with Slit2^22^. The vascular protective effect of Slit2 has been implicated to be dependent on Robo4. Slit2 was found to inhibit VEGF induced retinal hyperpermeability in Robo4+/+ mice but not in Robo4 null mice suggesting that Slit2-dependent inhibition of VEGF induced permeability was mediated via Robo4 activation^22^. Likewise, VEGF induced vascular permeability was attenuated by Slit2 in Robo4+/+ but not in Robo4−/− endothelial cells^49^. Likewise, Slit2 reduced Andes virus induced pulmonary microvascular endothelial cell permeability dependent on Robo4 *in vitro* ^48^. In accordance with previous studies, SBI rats that received recombinant Slit2 had decreased extravasation of albumin-bound Evans blue dye into peri-resection brain tissue post-SBI suggesting that Slit2 protected against SBI-induced BBB hyperpermeability.

The BBB interendothelial junction proteins are important components of BBB that maintain integrity of the BBB^50^. Tight junction proteins such as occludin and claudin3 maintain barrier function of the BBB, and disruption of tight junction proteins increases endothelial barrier permeability after an insult to the brain^1, 51^. Various factors such as inflammation, oxidative stress and proteases can degrade tight junction proteins after brain injury^51, 52^. Previous studies show that tight junction proteins were degraded in the perisurgical site thereby increasing BBB permeability and consequently worsening brain edema in SBI rodent models^1, 10^. The endothelial protective function of Slit2 was shown to be associated with stabilization of inter-endothelial junctions *in vitro* ^48^. In addition, transgenic overexpression of Robo4 by glioma cocultured endothelial cells reduced BTB permeability by preserving tight junction protein expression *in vitro* ^21^. We observed that recombinant Slit2 administration partially increased the expression of tight junction proteins after SBI suggesting that Slit2 regulates the expression of endothelial tight junction proteins. However, we did not observe any change in expression of adherens junction protein VE cadherin 24 hours after SBI with or without Slit2 administration.

The small GTPase protein Rac1 is an important mediator known to stabilize tight junction and adherens junction complexes that maintain endothelial barrier integrity^35, 53^. Robo4 overexpression in endothelial cells was shown to activate Rac1 *in vitro* whereas, knockdown of Robo4 reduced Rac activation^54^. We speculated that recombinant Slit2 binds to Robo4 receptors on endothelial cells and regulates the expression of tight junction proteins possibly by affecting Rac1 activation. We therefore investigated whether Slit2 regulates Rac1 activity dependent on Robo4. We examined the role of Robo4 and its downstream mediator Paxillin in recombinant Slit2 mediated regulation of Rac1 activity. The Paxillin Interaction Motif (PIM) in the cytoplasmic tail of Robo4 directly interacts with Lim domain of Paxillin^24^, and this interaction is increased when Slit2 is present. There are at least two possible ways by which Paxillin can initiate Rac1 activation. First, Paxillin can recruit Rac-specific GEF, bPIX which increases Rac1 activity^45^. Second, binding of Slit2 ligand to Robo4 increases the interaction of Robo4 with Paxillin and Arf-GAP complex^24^. The Arf-GAP complex inactivates Arf6 which is a small GTPase protein that induces the internalization of Rac1^55, 56^. In the presence of Slit2, increased interaction between Robo4-Paxillin inactivates Arf6 thereby restoring Rac1 activity. In accordance, we observed that recombinant Slit2 administration increased Rac1 activity after SBI which was reversed with siRNA knockdown of either Robo4 or Paxillin. Based on these findings, we infer that Slit2 preserved BBB integrity after SBI possibly by Robo4-Paxillin mediated activation of Rac1 which partly stabilized the tight junction proteins. Of note, we observed that Paxillin knockdown appeared to influence Robo4 expression after SBI although it was not significant. The off-target effects of siRNAs cannot be completely ruled out. However, overall the knockdown of either Robo4 or Paxillin reversed Rac1 activity increased by recombinant Slit2 suggesting that Robo4 and Paxillin were involved in Rac1 activation with recombinant Slit2.

Our study had some limitations. First, we did not provide direct evidence to show that Robo4 and Paxillin interaction increases upon recombinant Slit2 administration. Previous study showed that the interaction between Paxillin and Robo4 receptor is increased by Slit2^24^. Although we do not have direct evidence to show Robo4 and Paxillin interaction increased Rac1 activity upon Slit2 administration, our results showed that recombinant Slit2 administration increased Rac1 activity which was reversed with Robo4 and Paxillin knockdown. This suggests that Robo4 and Paxillin played a role in Rac1 activation in the presence of recombinant Slit2. Second, Rac1 may be among one of the factors that regulate BBB stability after SBI. Therefore, despite an increase in Rac1 activity with recombinant Slit2 administration, BBB junction stability and leakage was partly reversed after SBI. Third, it is possible that Robo4 may modulate BBB integrity through inhibition of matrixmetalloproteinase-9 (MMP-9) activity. Previous study showed that knockdown of Robo4 increased BTB permeability by upregulating MMP-9 in glioma endothelial cells *in vitro* ^21^. It is possible that recombinant Slit2 modulates MMP activity dependent on Robo4 to preserve BBB junction integrity. Additionally, other downstream molecules may be involved in Robo4 signaling pathway. For instance, the phosphorylation of Src and Erk1/2 were upregulated with knockdown of Robo4 *in vitro* ^21^. Likewise, abelson tyrosine kinase (Abl) and its substrate enabled (Ena) interact with the cytoplasmic tail of Robo4^14, 49^. Although, we did not evaluate alternate pathways, it is possible that Robo4 may modulate various downstream factors including Paxillin and Rac1 that may have contributed to the endothelial protective effects of recombinant Slit2. Lastly, we did not evaluate effects of Slit2 on other cell types apart from the BBB protective function. Recombinant Slit2 possibly crosses the BBB once it is disrupted following SBI and can therefore exert effects on other cell types in the brain including neurons that have been reported to express Robo receptors. In this study our objective was to evaluate the BBB protective effects of Slit2, therefore Slit2 was administered by intraperitoneal systemic route and the extravascular effects were not evaluated. We hypothesized that recombinant Slit2 interacts with endothelial Robo4 receptors thereby protecting against BBB permeability. Further studies are required to investigate the extravascular function of Slit2.

Overall, this study showed that recombinant Slit2 reduced BBB permeability by stabilizing endothelial tight junction proteins after experimental SBI possibly via Robo4-Paxillin dependent Rac1 activation.

## Electronic supplementary material

Supplementary Information

## References

[1] Jadhav V, Matchett G, Hsu FP, Zhang JH (2007). Inhibition of Src tyrosine kinase and effect on outcomes in a new *in vivo* model of surgically induced brain injury. J. Neurosurg..

[2] Frontczak-Baniewicz M, Walski M, Madejska G, Sulejczak D (2009). MMP2 and MMP9 in immature endothelial cells following surgical injury of rat cerebral cortex-a preliminary study. Folia Neuropathol..

[3] Houkin K (2009). Quantitative analysis of adverse events in neurosurgery. Neurosurgery..

[4] Huang KF, Hsu WC, Chiu WT, Wang JY (2012). Functional improvement and neurogenesis after collagen-GAG matrix implantation into surgical brain trauma. Biomaterials..

[5] Bruder N, Ravussin P (1999). Recovery from anesthesia and postoperative extubation of neurosurgical patients: a review. J. Neurosurg. Anesthesiol..

[6] Bruder NJ (2002). Awakening management after neurosurgery for intracranial tumours. Curr. Opin. Anesthesiol.

[7] Rolston JD, Han SJ, Lau CY, Berger MS, Parsa AT (2014). Frequency and predictors of complications in neurological surgery: national trends from 2006 to 2011. J. Neurosurg..

[8] Wong JM (2012). Patterns in neurosurgical adverse events: intracranial neoplasm surgery. Neurosurg. Focus..

[9] Jadhav, V., Solaroglu, I., Obenaus, A., Zhang, J. H. Neuroprotection against surgically induced brain injury. *Surg. Neurol*. **67**, 15–20, discussion 20 (2007b). 10.1016/j.surneu.2006.07.014 PMC1852449 17210286

[10] Yamaguchi, M., Jadhav, V., Obenaus, A., Colohan, A., Zhang, J. H. Matrix metalloproteinase inhibition attenuates brain edema in an *in vivo* model of surgically-induced brain injury. *Neurosurgery* **61**, 1067–1075, discussion 1075–1066 (2007). 10.1227/01.neu.0000303203.07866.18 18091283

[11] Nag S, Kapadia A, Stewart DJ (2011). Review: molecular pathogenesis of blood-brain barrier breakdown in acute brain injury. Neuropathol. Appl. Neurobiol..

[12] Li Q, Xu M, Zhou JX (2014). Correlation of measured and calculated serum osmolality during mannitol or hypertonic saline infusion in patients after craniotomy: a study protocol and statistical analysis plan for a randomised controlled trial. BMJ Open..

[13] Xu FF (2014). Effects of progesterone vs. dexamethasone on brain oedema and inflammatory responses following experimental brain resection. Brain Inj..

[14] Ballard MS, Hinck L (2012). A roundabout way to cancer. Adv. Cancer Res..

[15] Yuen DA, Robinson LA (2013). Slit2–Robo signaling: a novel regulator of vascular injury. Curr. Opin. Nephrol. Hypertens..

[16] Marillat V (2002). Spatiotemporal expression patterns of slit and robo genes in the rat brain. J. Comp. Neurol..

[17] Hagino S (2003). Slit and glypican-1 mRNAs are coexpressed in the reactive astrocytes of the injured adult brain. Glia..

[18] Sherchan P (2016). Recombinant Slit2 attenuates neuroinflammation after surgical brain injury by inhibiting peripheral immune cell infiltration via Robo1-srGAP1 pathway in a rat model. Neurobiol. Dis..

[19] Blockus H, Chedotal A (2016). Slit-Robo signaling. Development.

[20] Huminiecki L, Gorn M, Suchting S, Poulsom R, Bicknell R (2002). Magic roundabout is a new member of the roundabout receptor family that is endothelial specific and expressed at sites of active angiogenesis. Genomics.

[21] Cai H (2015). Roundabout 4 regulates blood-tumor barrier permeability through the modulation of ZO-1, occludin, and claudin-5 expression. J. Neuropathol. Exp. Neurol..

[22] Jones CA (2008). Robo4 stabilizes the vascular network by inhibiting pathologic angiogenesis and endothelial hyperpermeability. Nat. Med..

[23] London, N. R. *et al*. Targeting Robo4-dependent Slit signaling to survive the cytokine storm in sepsis and influenza. *Sci. Transl. Med*. **2**, 23ra19 (2010). 10.1126/scitranslmed.3000678 PMC2875996 20375003

[24] Jones CA (2009). Slit2-Robo4 signalling promotes vascular stability by blocking Arf6 activity. Nat. Cell Biol..

[25] Altay T, McLaughlin B, Wu JY, Park TS, Gidday JM (2007). Slit modulates cerebrovascular inflammation and mediates neuroprotection against global cerebral ischemia. Exp. Neurol..

[26] Suzuki H, Hasegawa Y, Kanamaru K, Zhang JH (2010). Mechanisms of osteopontin-induced stabilization of blood-brain barrier disruption after subarachnoid hemorrhage in rats. Stroke..

[27] Chen S (2013). P2X7R/cryopyrin inflammasome axis inhibition reduces neuroinflammation after SAH. Neurobiol. Dis..

[28] Garcia, J. H., Wagner, S., Liu, K. F., Hu, X. J. Neurological deficit and extent of neuronal necrosis attributable to middle cerebral artery occlusion in rats. Statistical validation. *Stroke* **26**, 627–634, discussion 635 (1995). 10.1161/01.str.26.4.627 7709410

[29] Ostrowski RP, Colohan AR, Zhang JH (2005). Mechanisms of hyperbaric oxygen-induced neuroprotection in a rat model of subarachnoid hemorrhage. J. Cereb. Blood Flow Metab..

[30] Soejima Y (2012). Hyperbaric oxygen preconditioning attenuates hyperglycemia enhanced hemorrhagic transformation after transient MCAO in rats. Med. Gas Res..

[31] Xu Q, Qaum T, Adamis PA (2001). Sensitive Blood–Retinal Barrier Breakdown Quantitation Using Evans Blue. Inves. Opthal. Vis. Sci..

[32] Li L (2015). G-CSF attenuates neuroinflammation and stabilizes the blood-brain barrier via the PI3K/Akt/GSK-3β signaling pathway following neonatal hypoxia-ischemia in rats. Exp. Neurol..

[33] Hasegawa Y, Suzuki H, Altay O, Zhang JH (2011). Preservation of tropomyosin-related kinase B (TrkB) signaling by sodium orthovanadate attenuates early brain injury after subarachnoid hemorrhage in rats. Stroke.

[34] Huang L (2015). Phosphoinositide 3-kinase gamma contributes to neuroinflammation in a rat model of surgical brain injury. J. Neurosci..

[35] Wojciak-Stothard B, Ridley AJ (2002). Rho GTPases and the regulation of endothelial permeability. Vascul. Pharmacol..

[36] Raz L (2010). Role of Rac1 GTPase in NADPH oxidase activation and cognitive impairment following cerebral ischemia in the rat. PLoS One.

[37] Wu JY (2001). The neuronal repellent Slit inhibits leukocyte chemotaxis induced by chemotactic factors. Nature.

[38] Nguyen-Ba-Charvet KT, Chedotal A (2002). Role of Slit proteins in the vertebrate brain. J. Physiol. Paris..

[39] Park JH, Pak HJ, Riew TR, Shin YJ, Lee MY (2016). Increased expression of Slit2 and its receptors Robo1 and Robo4 in reactive astrocytes of the rat hippocampus after transient forebrain ischemia. Brain Res..

[40] Seth P (2005). Magic roundabout, a tumor endothelial marker: expression and signaling. Biochem. Biophys. Res. Commun..

[41] Fu P (2015). c-Abl mediated tyrosine phosphorylation of paxillin regulates LPS-induced endothelial dysfunction and lung injury. Am. J. Physiol. Lung Cell. Mol. Physiol..

[42] Turner CE (2000). Paxillin and focal adhesion signalling. Nat. Cell Biol..

[43] Fu P (2015). Role played by paxillin and paxillin tyrosine phosphorylation in hepatocyte growth factor/sphingosine-1-phosphate-mediated reactive oxygen species generation, lamellipodia formation, and endothelial barrier function. Pulm. Circ..

[44] Turner CE (2000). Paxillin interactions. J. Cell Sci..

[45] Birukova AA, Cokic I, Moldobaeva N, Birukov KG (2009). Paxillin is involved in the differential regulation of endothelial barrier by HGF and VEGF. Am. J. Respir. Cell Mol. Biol..

[46] Ayer RE (2012). Preoperative mucosal tolerance to brain antigens and a neuroprotective immune response following surgical brain injury. J. Neurosurg..

[47] Zhang X (2012). Slit2/Robo4 signaling modulates HIV-1 gp120-induced lymphatic hyperpermeability. PLoS Pathog..

[48] Gorbunova EE, Gavrilovskaya IN, Mackow ER (2013). Slit2-Robo4 receptor responses inhibit ANDV directed permeability of human lung microvascular endothelial cells. Antiviral Res..

[49] Park KW (2003). Robo4 is a vascular-specific receptor that inhibits endothelial migration. Dev. Biol..

[50] Nag S, Manias JL, Stewart DJ (2009). Pathology and new players in the pathogenesis of brain edema. Acta Neruopathol..

[51] Altay O (2012). Isoflurane attenuates blood-brain barrier disruption in ipsilateral hemisphere after subarachnoid hemorrhage in mice. Stroke.

[52] Krafft PR (2013). PHA-543613 preserves blood-brain barrier integrity after intracerebral hemorrhage in mice. Stroke.

[53] Waschke J (2004). Requirement of Rac activity for maintenance of capillary endothelial barrier properties. Am. J. Physiol. Heart and Circ. Physiol..

[54] Kaur S (2006). Robo4 signaling in endothelial cells implies attraction guidance mechanisms. J. Biol. Chem..

[55] Palacios F, Price L, Schweitzer J, Collard JG, D’Souza-Schorey C (2001). An essential role for ARF6-regulated membrane traffic in adherens junction turnover and epithelial cell migration. EMBO J.

[56] Turner CE, West KA, Brown MC, Paxillin-ARF GAP (2001). signaling and the cytoskeleton. Curr. Opn. Cell Biol..

