# Supplementary material for: Recombinant Slit2 Reduces Surgical Brain Injury Induced Blood Brain Barrier Disruption via Robo4 Dependent Rac1 Activation in a Rodent Model
Source: Sci Rep. 2017 Apr 7;7:746. doi: 10.1038/s41598-017-00827-z (PMC5429690; doi:10.1038/s41598-017-00827-z)
Supplement: Supplementary file 1 — Supplementary Information [file 41598_2017_827_MOESM1_ESM.doc]

**Supplementary Information**

**Recombinant Slit2 Attenuates Surgical Brain Injury Induced Blood Brain Barrier Disruption via Robo4 Dependent Rac1 Activation in a Rat Model**

Prativa Sherchan, MBBS, PhD^a^, Lei Huang, MD^a,b^, Onat Akyol, MD^a^, Cesar Reis, MD^a^, Jiping Tang, MD^a^, John H. Zhang, MD, PhD^a,b^**^*^**

^a^Department of Physiology and Pharmacology, Loma Linda University, Loma Linda, California, 92354, U.S.A.; ^b^Department of Anesthesiology, Loma Linda University, California, 92354, U.S.A.

***Corresponding Author**:

John H. Zhang, MD, PhD

Departments of Anesthesiology, Neurosurgery and Physiology

Loma Linda University, School of Medicine

Loma Linda, CA 92354, U.S.A.

Phone: (909) 558-4723, Fax: (909) 558-0119, Email: [johnzhang3910@yahoo.com](mailto:johnzhang3910@yahoo.com)

**Source of Funding**: This study was partially supported by NIH NS084921 to JHZ.

**Conflict of Interest Statement:** The authors have declared that no conflict of interest

exists.

**Supplementary Figure S1.**

**(A)** **
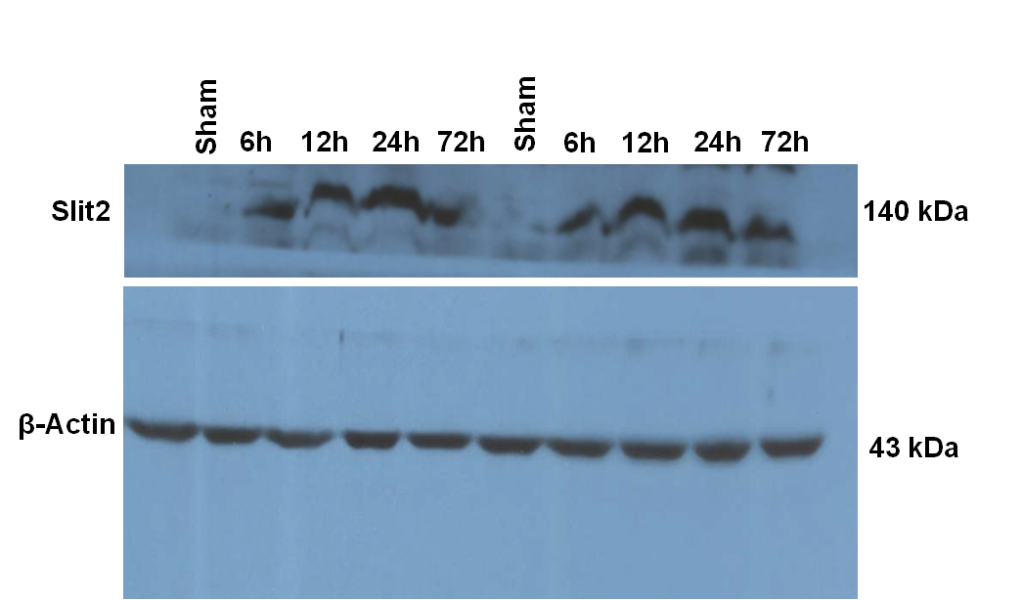
**

**(B)**
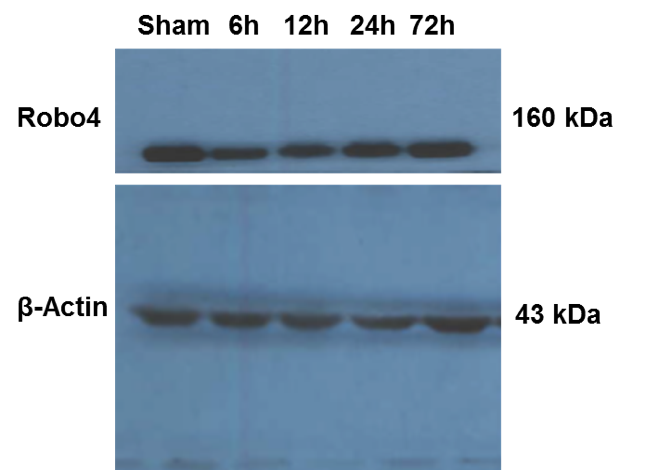


**(C)**
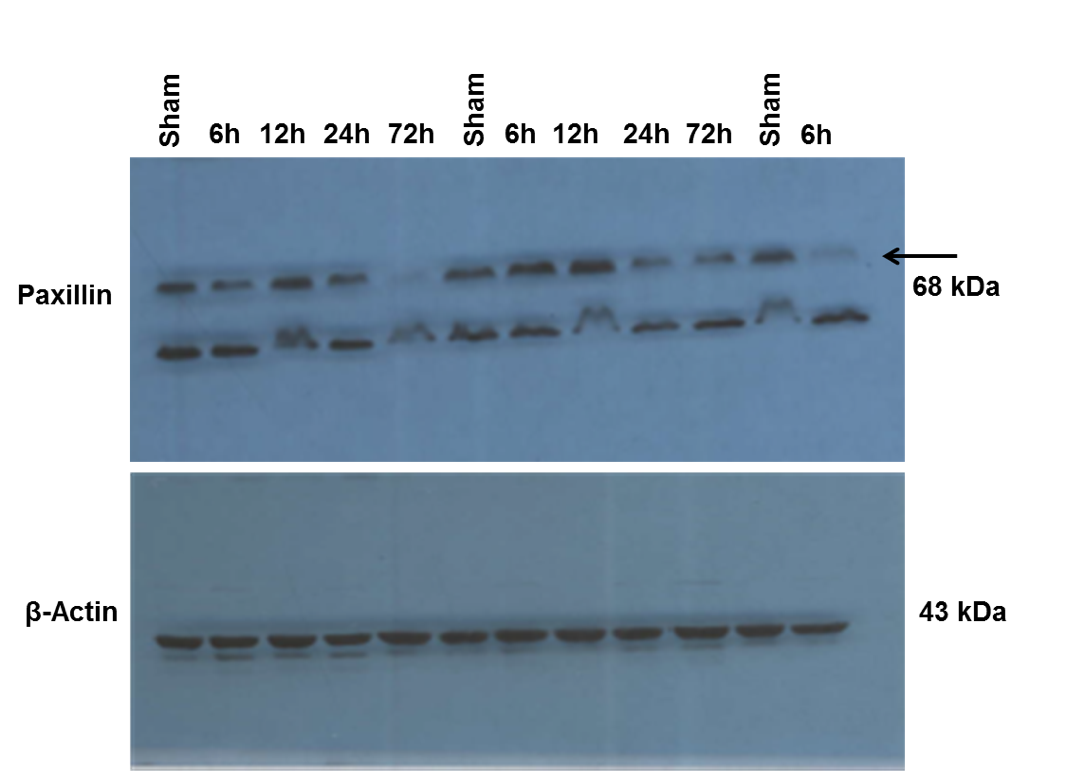


**Supplementary Figure S1.** Full length blots for representative western blot pictures shown in Figure 1. Each lane was loaded with sample from a single animal from the group that is labeled in the blot. The same membrane was probed for β-actin as loading control. Temporal expression of Slit2 **(A)**, temporal expression of Robo4 **(B)**, temporal expression of Paxillin **(C)** was evaluated at 6h, 12h, 24h, and 72h after SBI.

**Supplementary Figure S2.**

**(A)
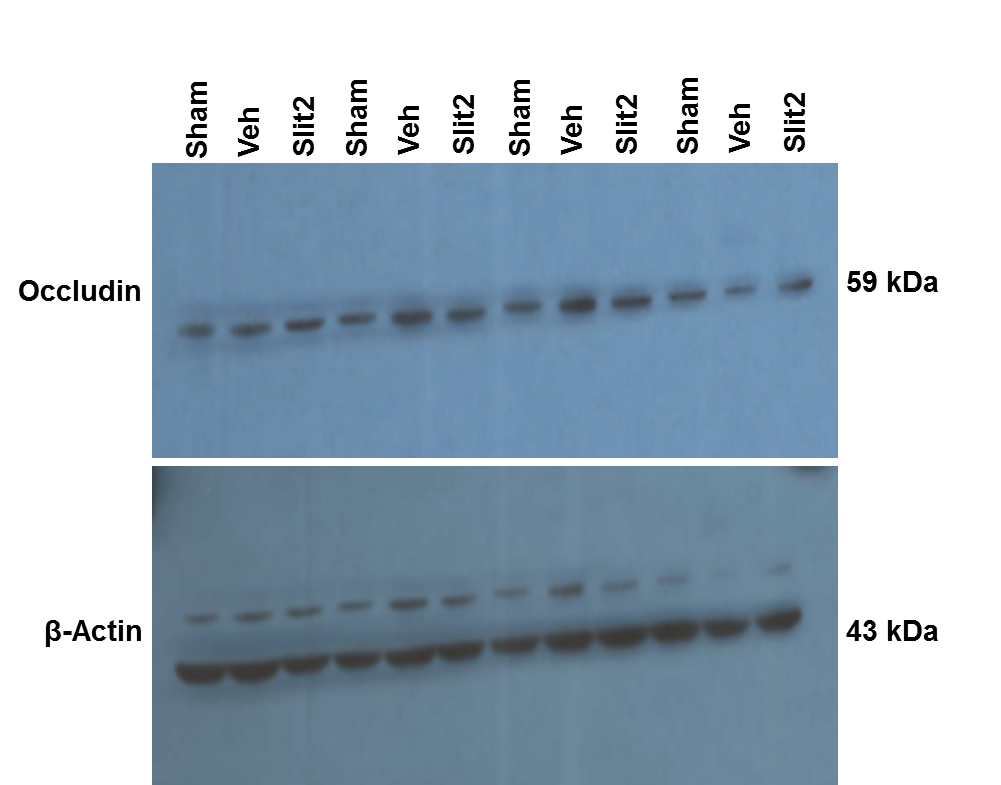
**

**(B)
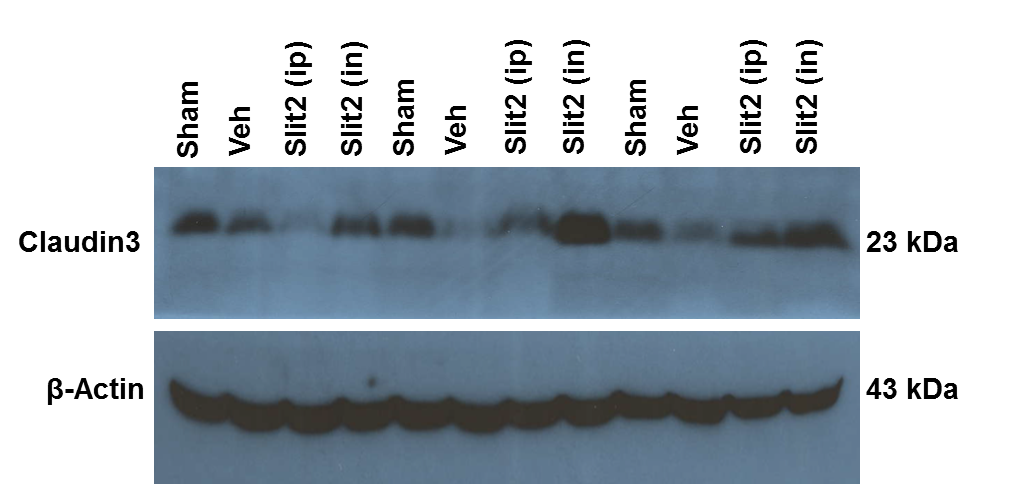
**

**(C)
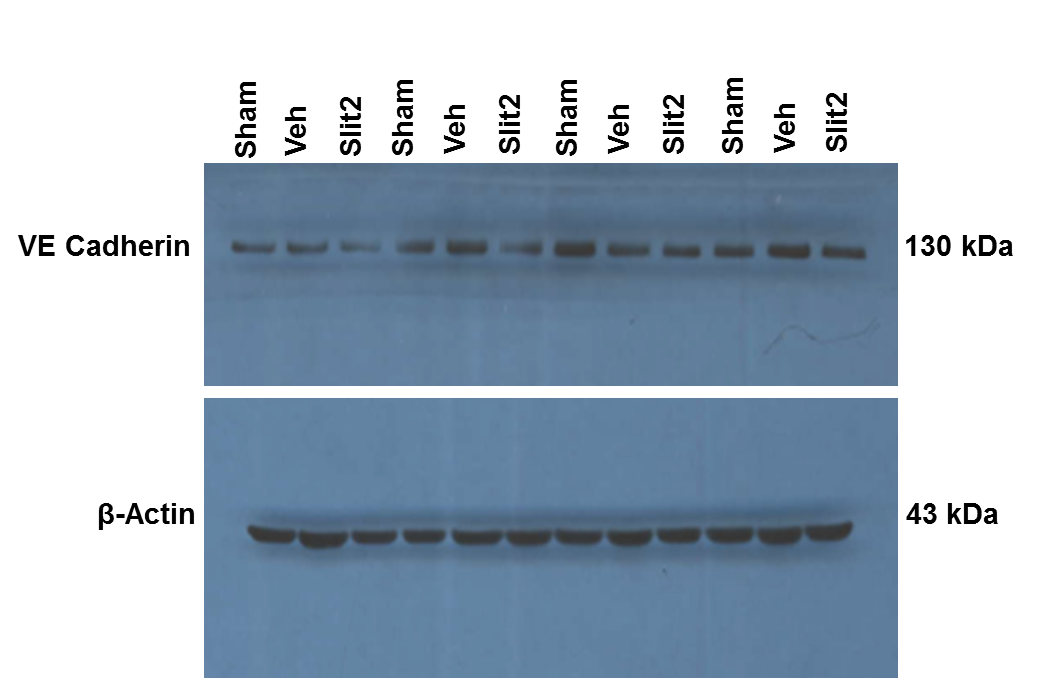
**

**Supplementary Figure S2.** Full length blots for representative western blot pictures shown in Figure 4. Each lane was loaded with sample from a single animal from the group that is labeled in the blot. The same membrane was probed for β-actin as loading control. Expression of BBB junction proteins occludin **(A),** claudin 3 **(B)**, and VE cadherin **(C)** was evaluated 24h after SBI with or without recombinant Slit2 (10 μg/Kg) administration 1h before injury. In the blot on panel C, a group of animals that received recombinant Slit2 (10 μg/Kg) administered via intranasal route were also run on the same gel. Abbreviations: Veh (Vehicle), Slit2 (recombinant Slit2 10 μg/Kg), ip (intraperitoneal route), in (intranasal route).

**Supplementary Figure S3.**

**
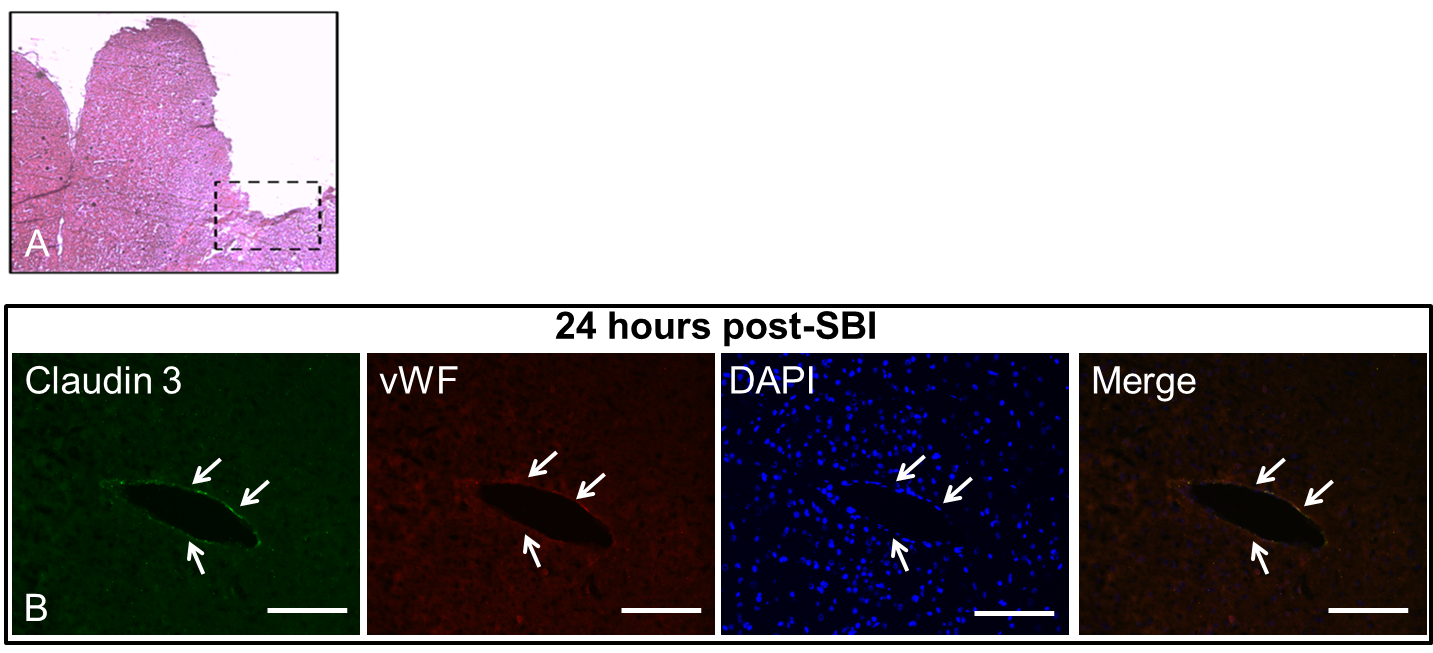
**

**Supplementary Figure S3.** **(A)** Brain section of an SBI rat showing location in the inset used for taking pictures for immunohistochemistry. **(B)** Localization of Claudin 3 in the brain at the right frontal perisurgical site 24h after SBI. Microphotograph of immunofluorescence staining showed co-localization of Claudin 3 (FITC/green) with the endothelial marker von Willibrand Factor (vWF) (Rhodamine Red/red) and DAPI (blue). Scale bar=100μm.

**Supplementary Figure S4.**


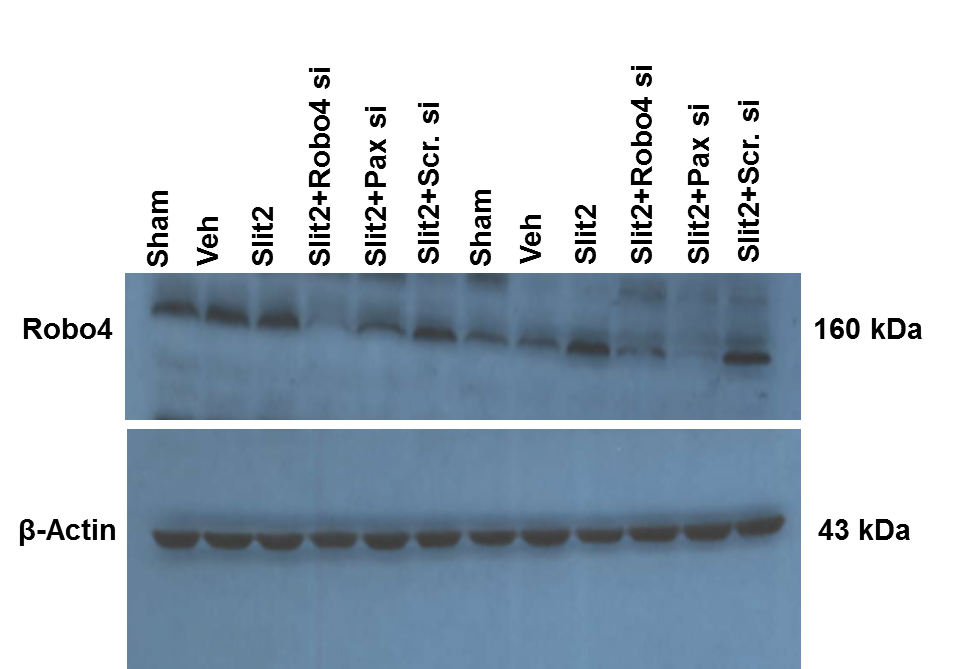


**(A)**


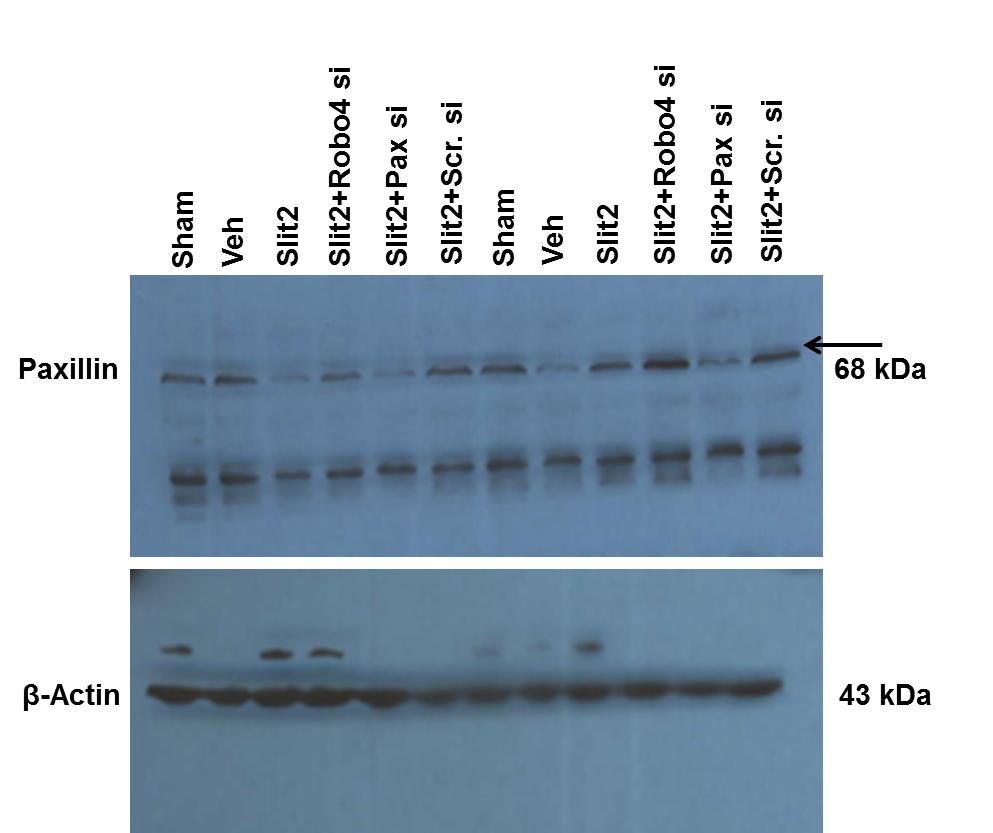


**(B)**

**
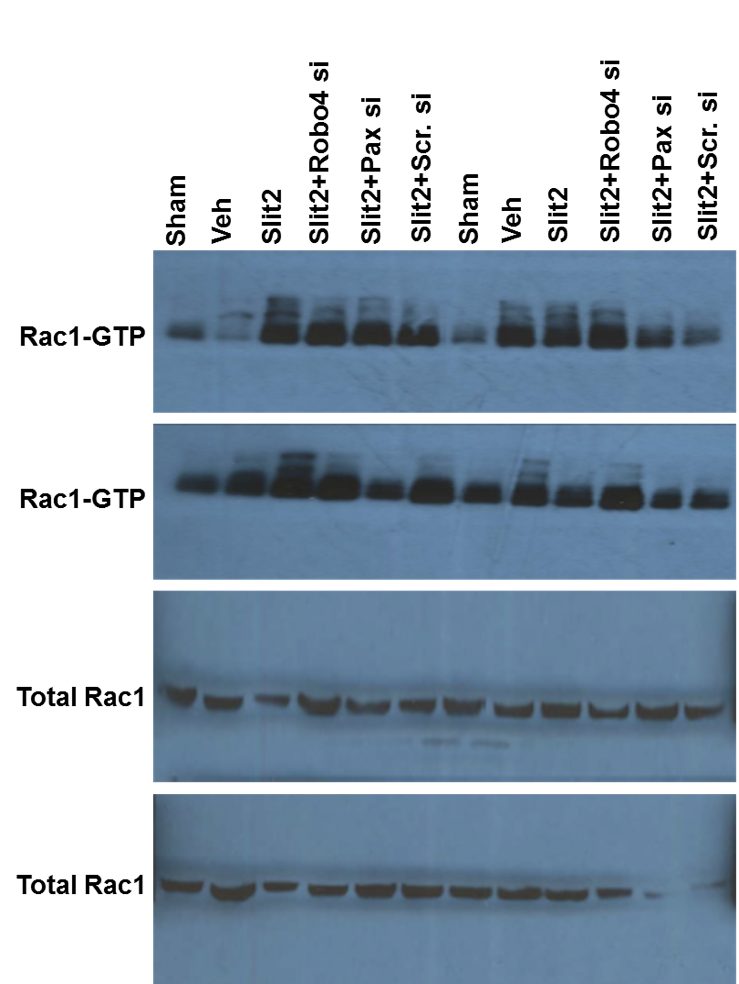
**

**(C)**

**Supplementary Figure S4.** Full length blots of western blot pictures **(A, B)** and Rac1 activity assay **(C)** for pictures shown in Figure 6. The expression of Robo4 **(A)** and Paxillin **(B)** was evaluated 24h after SBI with or without recombinant Slit2 (10 μg/Kg) administration 1h before injury. The siRNA administered groups received the respective siRNAs 24h before SBI. Each lane was loaded with sample from a single animal from the group that is labeled in the panels in the figure. The same membrane was probed for β-actin as loading control. Rac1 activity assay **(C)** was evaluated 24h after SBI with or without Slit2 (10 μg/Kg) administration 1h before injury. Each lane was loaded with sample from a single animal from the group as labeled. Abbreviations: Veh (Vehicle), Slit2 (recombinant Slit2 10 μg/Kg), Robo4 si(Robo4 siRNA), Pax si (Paxillin siRNA), Scr. Si (Scramble siRNA).

**Supplementary Table S1: Western blot antibodies list**

| Antibody catalog # | Vendor | Target | Application | Reactivity | Host |
| --- | --- | --- | --- | --- | --- |
| sc-16619 | Santacruz Biotechnology | Slit2 | WB, IF, ELISA | Mouse, rat, human | Goat |
| sc-166872 | Santacruz Biotechnology | Robo4 | WB, IHC, IF, ELISA | Mouse, rat, human | Mouse |
| sc-7336 | Santacruz Biotechnology | Paxillin | WB, IHC | Mouse, rat, human | Goat |
| ab167161 | Abcam | Occludin | WB, IP | Mouse, rat, human | Rabbit |
| ab15102 | Abcam | Claudin 3 | WB, IHC | Mouse, rat, human | Rabbit |
| sc-6458 | Santacruz Biotechnology | VE Cadherin | WB, IF, ELISA | Mouse, rat, human | Goat |
| sc-1616 | Santacruz Biotechnology | Actin | WB, IF, IHC, ELISA | Mouse, rat, human | Goat |
